# Shared decision-making in post-coercion debriefing interventions in psychiatry – a scoping review

**DOI:** 10.3389/fpsyt.2024.1446619

**Published:** 2024-09-27

**Authors:** Katharina Froelich, Jan Schürmann, Christian G. Huber, Manuel Trachsel

**Affiliations:** ^1^ Clinical Ethics Unit, University Hospital Basel (USB), University Psychiatric Clinics (UPK) Basel, Geriatric University Hospital FELIX PLATTER Basel (UAFP), and University Children’s Hospital Basel (UKBB), Basel, Switzerland; ^2^ Medical Faculty, University of Basel, Basel, Switzerland; ^3^ Clinic for Adult Psychiatry, University Psychiatric Clinics (UPK) Basel, Basel, Switzerland

**Keywords:** coercion, debriefing, shared decision-making, psychiatry, decision aids, scoping review

## Abstract

**Introduction:**

Debriefing is recommended after any coercive measure in psychiatry, but there are no wellestablished standards, and ist effectiveness remains unclear. Incorporating shared decision-making (SDM) into post-coercion debriefing interventions has potentially beneficial effects.

**Methods:**

This scoping review provides an overview of the general characteristics of such interventions and the extent to which SDM elements are already used in such interventions.

**Results:**

A total of 2562 references were identified in the scholarly databases Embase, PubMed, Web of Science, and PsycINFO. In addition, 14 articles were identified through manual searches of reference lists. 42 full-text articles were screened for eligibility, 13 articles met the eligibility criteria and were further analyzed.

**Discussion:**

No intervention tool was found that clearly included all SDM elements. However, three elements of SDM were present at least partially in all interventions: definition and explanation of the health care problem, the clarification of the patient's values and preferences, and a decision or explicit deferral of the decision. Further research is needed to systematically examine the implementation and clinical effectiveness of post-coercion debriefing interventions, particularly regarding the inclusion of shared decision-making elements.

## 1 Introduction

In Switzerland, 10.4% of all adult patients in acute and primary psychiatric care were affected by a formal coercive measure such as seclusion, restraint, or forced medication in 2022 (1). Coercive measures are defined as any measure “carried out against the patient’s self-determined wishes or in spite of his or her opposition” (2). They can be a significant burden for patients, relatives and health care providers (3–5). Moreover, the use of coercion contradicts the ethical principle of respect for patient autonomy and should therefore only be used when necessary and appropriate to prevent a serious risk to self or others (6). The elimination of unjustified coercion and the reduction of coercion in psychiatry is a goal of several national and transnational guidelines (7, 8).

Debriefing with patients after coercive measures is one intervention among several others to achieve this goal (9). A debriefing should reflect on the situation that led to the use of coercive measures and aim at a retrospective understanding of the different perspectives of all those involved. This should provide emotional relief and transparency and strengthen the therapeutic relationship (10). Medical ethical guidelines in Switzerland and Germany recommend that a debriefing should take place after every coercive measure (2, 8). However, no standards have yet been established for the content, implementation, and conduct of debriefings (10). Only a few projects have systematically implemented post-coercion debriefings, mostly as part of more complex programs such as the Weddinger Model (11) or the Six Core Strategies (12). To date, there is little research on the effectiveness of debriefing as a stand-alone intervention to prevent future coercion (13, 14).

In addition to a retrospective reflection on the coercive event, debriefing can emphasize prevention by allowing strategies to be developed to avoid or reduce the impact of coercive measures in the future (15). Hereby, insights from the retrospective part of the debriefing can be used to inform future care plans (16). Shared decision-making (SDM) can be considered as one approach to shape and conduct this prospective part of the post-coercion debriefing intervention. This involves health care professionals and patients discussing available options together and choosing the option that best fits the patient’s circumstances and preferences (17). Patient-centered care, which emphasizes participation, recovery, and transparency, advocates SDM as the preferred form of medical decision making (18, 19). However, there is currently no established standard approach to SDM in psychiatry (20), and most approaches focus on psychopharmacological decisions and therefore do not address the complex realities of psychiatric patients (21).

There is growing evidence of the beneficial effects of using SDM in psychiatric interventions. SDM interventions have been shown to be effective for reducing involuntary admissions (22, 23). Recently, an SDM intervention for the first week after involuntary admission was developed and found to be feasible and well received by patients and treatment providers (24). Another study found that an SDM intervention for patients admitted to a mental health facility against their will improved patients’ perceived involvement in decision making in a manner similar to that observed for those admitted voluntarily (25). However, the intervention, which did not specifically target decision making in the context of a coercive event, did not reduce aggressive incidents or coercive measures. Most patients want to be involved in decisions about restraint (26). Involvement of involuntary patients is also a strong predictor of their subjective quality of life and treatment satisfaction for these patients (27). Facilitating and confounding factors of SDM in involuntarily admitted patients have also been investigated (28).

Decision aids (DAs) are important tools for translating the principles of SDM into clinical and practical reality (29). Digitalization provides an opportunity to design DAs in an interactive, personalized, and potentially more effective manner (30). Digital DAs have promising practical applications in psychiatry to improve patient motivation and the therapeutic relationship, and to reduce decision-making conflict (31). Therefore, digital DAs may have the potential to facilitate SDM in post-coercion debriefing in psychiatry.

The findings of this scoping review will inform the development of a digital DA to support clinical practitioners in implementing SDM in post-coercion debriefing interventions in psychiatry. Previous research does not explicitly report on SDM interventions that were used in this context. Krieger and colleagues (10) conducted a narrative review of post-coercion debriefing but provided limited information on specific tools and their characteristics. Therefore, a systematic search for literature on SDM elements in debriefing interventions in the described context is considered as necessary. The aim of this scoping review is to identify the range of SDM elements used in post-coercion debriefing interventions in psychiatry. In addition, an analysis of general characteristics will provide further insight into the practical application and the requirements of these interventions. This scoping review addresses the following two research questions:

1. What SDM elements are included in post-coercion debriefing interventions in psychiatry?
2. What general characteristics are found in post-coercion debriefing interventions in psychiatry?

## 2 Methods

We conducted the present scoping review in accordance with the JBI methodology for scoping reviews (32). The final version of the report follows the Preferred Reporting Items for Systematic review and Meta-analysis extension for Scoping Reviews (PRISMA-ScR) as proposed by Tricco and colleagues (33). The review protocol was not pre-registered.

### 2.1 Study eligibility

Eligible publications were peer-reviewed articles including original theoretical, qualitative, quantitative, multi-methods, and mixed-methods studies, as well as all evaluation study designs (pre-experimental, experimental, randomized controlled trials (RCTs), quasi-experimental). Unpublished or non-peer-reviewed articles were not included, as the reviewers expected high-quality results to be published in peer-reviewed journals. Articles in English, German and French were included based on the language skills of the research team. Articles published between 01/01/2013 and 13/07/2023 were included as no relevant literature was expected to be published before this date.

Articles were selected if they reported data on intervention tools with SDM elements following coercive measures in psychiatry. The target population was 18 years of age or older with a DSM or ICD psychiatric diagnosis and experience with coercive measures in psychiatry. All psychiatric settings for adult patients were included (inpatient and outpatient) such as acute or long-term adult psychiatric wards, geriatric psychiatric wards, psychiatric outpatient clinics, or forensic psychiatric wards. Somatic hospitals, retirement homes and nursing homes were excluded due to the specific nature of these settings.

Post-coercion debriefing interventions were broadly defined as any structured conversation between patient and health care professionals following a coercive event with the aim of enabling patients and staff to view the event from each other’s perspectives, repairing ruptures in the therapeutic alliance and strengthening working relationships, providing emotional expression and relief in relation to the experienced situation, or preventing the use of further coercive measures.

Studies were selected if they addressed the issue of future coercion and/or its prevention, in addition to reflecting on a past coercive event, and used at least two key elements of SDM in this intervention. The key elements of SDM were operationalized according to the established SDM model of Makoul and Clayman (17) (**Table 1**).

**Table 1:** Definitions for the key elements of SDM.

Key element of SDM	Definition for this study
1) Define/explain the healthcare problem	Explicit definition and/or explanation of the problem that needs to be addressed.
2) Present options	Review of existing options by the health care professional, patients should raise options of which they may be aware.
3) Discuss pros/cons (benefits, risks, costs)	Discussion of pros and cons of available options, particularly because there may be different perspectives on the relative importance of benefits, risks, and costs.
4) Clarify patient values/preferences	Clarification of patient values and preferences – including ideas, concerns, and outcome expectations.
5) Discuss patient ability/ self-efficacy	Discussion of patients’ ability, or self-efficacy, to follow through with a plan as a component of assessing the viability of options.
6) Professional knowledge/recommendations	Presentation of professional knowledge and recommendations in the context of the decision at hand.
7) Check/clarify understanding	Periodical check of understanding of facts and perspectives, providing further clarification as needed.
8) Make or explicitly defer decision	Decisions are explicitly recorded. OR Decisions may be explicitly deferred for a later time (e.g., pending discussion with members of the family and/or healthcare team).
9) Arrange follow-up	Arrangement on follow-up to track the outcome of decisions that have been made or reach resolution on those that have not.

Adapted from Makoul and Clayman (17); SDM = shared decision-making.

### 2.2 Information sources and search strategy

An initial limited search of PubMed and Cochrane was conducted to identify articles on the topic. In consultation with an academic librarian from the University Library of Basel, the text words contained in the titles and abstracts of relevant articles and the index terms used to describe the articles were used to develop full search strategies. For peer-reviewed literature, the databases Embase, Web of Science, PubMed, and PsycINFO were searched using search terms related to the concepts of psychiatry, coercion, intervention, and SDM. The search strategy, including all identified keywords and index terms, was adapted for each included database (see **Supplementary Material**). Search terms included Emtree in Embase, free text terms in Web of Science, MeSH in PubMed, and indexed terms from the thesaurus in PsycINFO.

### 2.3 Study selection

Following the search, all identified references were collated and uploaded into Citavi (version 6.17, Lumivero: Denver, USA) and then imported into the Covidence systemic review software (Veritas Health Innovation: Melbourne, Australia, 2023). After deduplication, two reviewers (JS and KF) independently screened all titles and abstracts against the inclusion criteria. Disagreements were resolved by a third reviewer (MT). In the next stage of the screening process, the full texts of the selected references were screened using the same procedure. Reasons for exclusion of references were recorded.

### 2.4 Data extraction

Data were extracted from each included article by two independent reviewers (JS and KF). Disagreements were resolved by discussion to reach consensus. An extraction form developed by the reviewers was used, which was modified and revised as necessary during the process. Data were extracted on bibliographic information (authors, title, year of publication, country), general study details (study design, sample size, setting, focus on specific disorders) and general characteristics of the intervention applied (name of intervention tool, participants and their roles, duration of the intervention, timepoint of application, training before use of tool). In addition, an in-depth qualitative analysis of the application of SDM elements in the intervention tools was conducted. We therefore used the definitions in **Table 1** to determine which key SDM elements were present. In two cases (13, 34) the authors of the articles were contacted to request additional data for this analysis, which we were unable obtain.

### 2.5 Data analysis and synthesis of results

Data on general elements of the articles were presented in tabular form. The results of the qualitative analysis of the SDM elements in the intervention tools were used to categorize each of the nine SDM elements as present or absent for each article in an additional table. The elements were marked as “unclear” if the interventions were not clearly described and/or if the reviewers were uncertain whether the relevant element was met in all aspects. All tabulated results are accompanied by a narrative summary describing how they relate to the objectives and questions of the scoping review.

## 3 Results

### 3.1 Study selection

**Figure 1:**
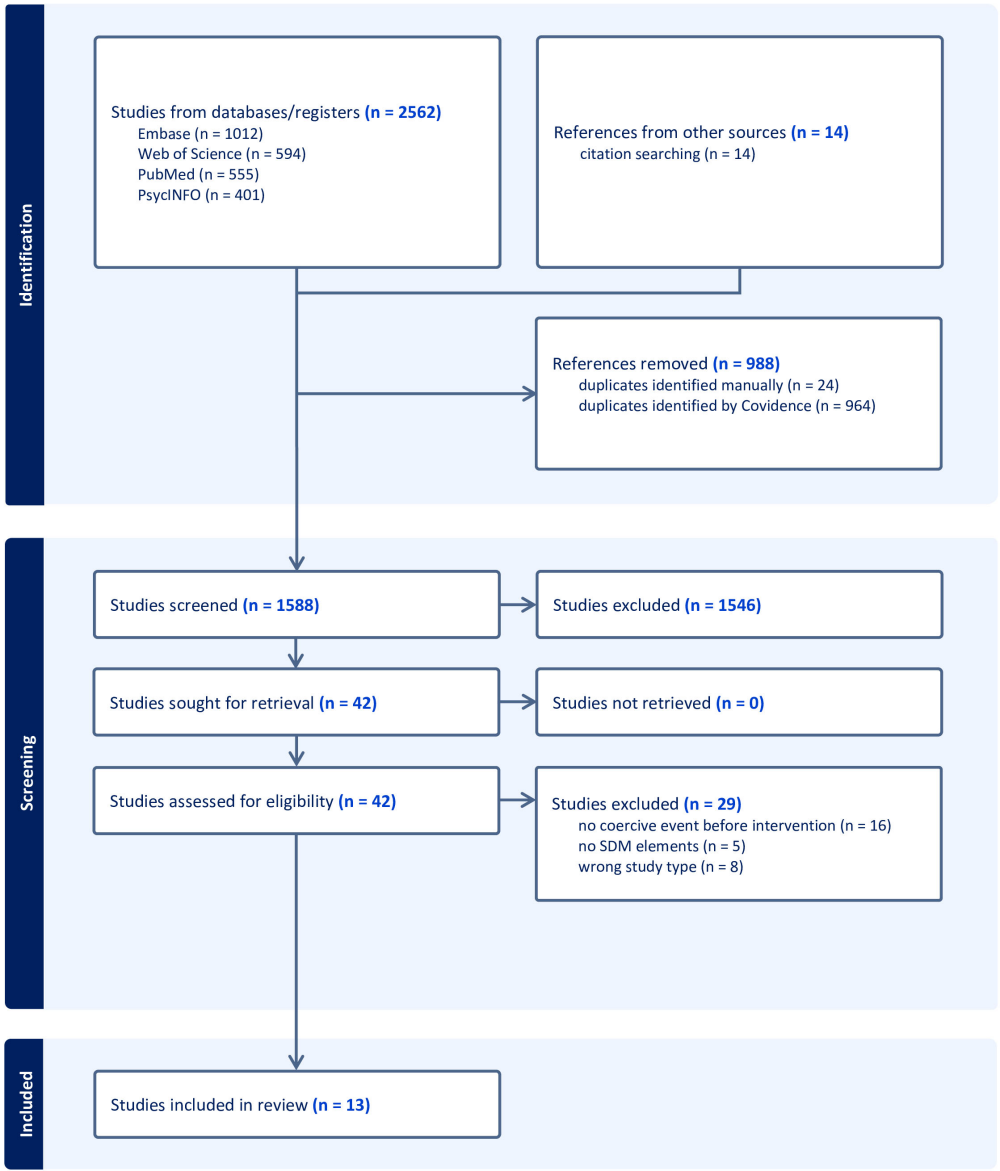
PRISMA flow diagram.

An overview of the study selection process is described in the PRISMA flow diagram (**Figure 1**). A total of 2562 references were identified in the selected scientific databases (Embase n = 1012; Web of Science n = 594; PubMed n = 555; PsycINFO n = 401). An additional 14 articles were identified by hand searching the reference lists of the identified articles.

After removing duplicates, 1588 references underwent title/abstract screening. 42 full-text articles were assessed for eligibility. We excluded 29 articles, most often because the intervention did not follow a coercive event (n = 16), did not include SDM elements (n = 5), or because the study design did not meet the inclusion criteria (n = 8). Thirteen articles met the eligibility criteria and were further analyzed.

### 3.2 Study details

**Table 2** shows the bibliographic information and study details of the 13 articles included in this scoping review. More than one third of the articles each used a qualitative study design (n = 5; 38%) or an experimental study design (randomized controlled trial n = 3; 23%; quasi-experimental n = 2; 15%). Two articles (15%) described interventions at a theoretical level. Most researchers or research teams were based in Europe (n = 10; 77%). More than half of the articles (n = 8; 62%) involved research with patients, some articles involved research with care providers (n = 3; 23%). A variety of settings were represented, with the most articles coming from acute inpatient settings (n = 6; 46%). As some settings were not described in detail, but only mentioned that the intervention took place in a hospital, we estimate this number to be higher. In two articles, the intervention also took place in a community mental health setting (n = 2; 15%). The majority of the articles focusing on specific psychiatric disorders included patients with psychotic disorders (n = 8; 62%). Other psychiatric diagnoses mentioned were affective disorders (n = 2; 15%) and substance use disorders (n = 2; 15%).

**Table 2:** Bibliographic information and study details of the included articles.

Authors, year	Country	Study design	Sample size	Setting	Focus on specific disorder(s)
Asikainen et al., 2023 (45)	Finland	chart review	524 (debriefing forms)	forensic in-patient psychiatric ward	psychotic disorders, substance abuse disorders, other
Dike et al., 2021 (32)	USA	quasi - experimental intervention study	n.a.	public sector psychiatry hospital	n.a.
Drack-Schönenberger et al., 2016 (46)	Switzerland	RCT	238 (119 intervention, 119 control)	psychiatric clinics, in-patient treatment	several non-specified psychiatric disorders
Freier, 2022 (39)	Switzerland	theoretical paper	0	n.a.	n.a.
Goulet et al., 2018 (40)	Canada	qualitative design	15 (12 care providers, 3 patients)	acute adult psychiatric care unit	psychotic disorders
Hammervold et al., 2020 (35)	Norway	qualitative design	19 (care providers)	university hospital, community mental health center	psychotic disorders, substance abuse disorders, affective disorders, other
Hammervold et al., 2022 (36)	Norway	qualitative design	8	university hospital, community mental health center	n.a.
Lorem et al., 2014 (42)	Norway	qualitative design	9	n.a.	psychotic disorders
Mahler et al., 2021 (15)	Germany	theoretical paper	0	n.a.	n.a.
Tinland et al., 2022 (47)	France	RCT	394 (196 intervention, 198 control)	mental health facilities (unspecified)	psychotic disorders
Whitecross et al., 2013 (13)	Australia	quasi - experimental intervention study	31 (17 intervention, 14 control)	acute in-patient psychiatric ward	psychotic disorders, affective disorders, other
Wullschleger et al., 2019 (37)	Germany	qualitative design, survey	27 (12 patients, 15 care providers)	acute in-patient psychiatric ward	psychotic disorders
Wullschleger et al., 2021 (38)	Germany	RCT	109 (52 intervention, 57 control)	acute in-patient psychiatric ward	psychotic disorders

other = other non-specified psychiatric disorders; RCT = randomized controlled trial; n.a. = not available.

### 3.3 General characteristics of the interventions

A selection of relevant general characteristics of the post-coercion interventions is shown in **Table 3**. Several articles described the same intervention (35, 36): and (15, 37, 38). The two theoretical articles applied the same rationale and general outline with slight adaptations of the research questions used (15, 39). One article additionally described a tool for a debriefing with caregivers involved in the coercive event, but without the presence of the patient (40).

**Table 3:** General characteristics of the identified post-coercion debriefing interventions.

Authors, year	Name of intervention tool	Participants and their roles	Duration of the intervention	Timepoint of application	Training before use of tool
Asikainen et al., 2023 (45)	Debriefing (Six Core Strategies)	patient, nurse	n.a.	n.a.	yes
Dike et al., 2021 (32)	Debriefing (Six Core Strategies)	n.a.	n.a.	n.a.	n.a.
Drack-Schönenberger et al., 2016 (46)	Crisis Card	patient, psychologist (part of the study team)	3-4 h (several meetings possible)	n.a.	n.a.
Freier, 2022 (39)	Gesprächsleitfaden zur Nachbesprechung von Zwangsmaßnahmen	patient, contact person of patient in the health care team, care provider involved in the restraint event, physician in charge	flexible	recommended up to 72 h after the coercive event	yes
Goulet et al., 2018 (40)	Tool 1 (patient): post-seclusion and/or restraint review (PSRR) following Bonner’s Model; Tool 2 (health care team): post-seclusion and/or restraint review (PSRR) following Huckshorn’s Model	Tool 1 (patient): patient, staff member present at coercive event; Tool 2 (health care team): health professionals involved at the event	10 - 30 min	Tool 1: 24–48 h after coercive event; Tool 2: one to several days after coercive event	yes
Hammervold et al., 2020 (35)	Post Incident Review (PIR)	Setting 1: patient, a milieu therapist who knew the patient, a responsible doctor or psychologist, the person responsible for the restraint decision and a relative based on the patient’s preferences; Setting 2: patient, moderation by a person not involved in the restraint incident, care provider involved in the restraint event	n.a.	Setting 1: as soon as possible and latest by discharge; Setting 2: as soon as possible after the restraint event, if possible not later than 72 h	n.a.
Hammervold et al., 2022 (36)	Post Incident Review (PIR)	Setting 1: patient, eventually next of kin, contact nurse or available familiar nurse and responsible therapist; Setting 2: patient, moderation by a person not involved in the restraint incident, care provider involved in the restraint event	n.a.	Setting 1: as soon as possible and latest by discharge; Setting 2: as soon as possible after the restraint event, if possible not later than 72 h	n.a.
Lorem et al., 2014 (42)	n.a.	n.a.	n.a.	n.a.	n.a.
Mahler et al., 2021 (15)	Leitfaden-gestützte Nachbesprechung von Zwangsmassnahmen im Weddinger Modell	patient, eventually next of kin/contact person of patient, team member not involved in the restraint incident (moderation), one care provider involved in the restraint event	30 - 45 min	patients themselves determine the preferred point of time	yes
Tinland et al., 2022 (47)	Psychiatric Advance Directive (PAD)	patient, peer worker	n.a. (as many meetings as necessary)	n.a.	n.a.
Whitecross et al., 2013 (13)	Post-seclusion counselling intervention	patient, nurse	n.a.	recommended to be carried out 3 days after the seclusion event, range: 3-7 days	yes
Wullschleger et al., 2019 (37)	Leitfadengestützte Nachbesprechung vonZwangsmaßnahmen im Weddinger Modell	patient, staff member actively involved in the coercion decision, any person of trust or another member of staff chosen by the patient, moderation by a member of staff not directly involved in the coercive event	30 - 40 min	patients themselves determine the preferred point of time, range: 1 - 118 days; mean: 39.9 days	yes
Wullschleger et al., 2021 (38)	post-coercion reflecting review session	see Wullschleger et al. (2019) (37)	see Wullschleger et al. (2019) (37)	see Wullschleger et al. (2019) (37)	see Wullschleger et al. (2019) (37)

other = other non-specified psychiatric disorders; RCT = randomized controlled trial; n.a. = not available.

The number of participants in the intervention ranged from the patient with one member of the study team to several participants with different roles. These included, for example, the patient’s next of kin, the patient’s contact person in the health care team, care providers involved in the coercive event, or other responsible care providers. Almost half of the articles (n = 6; 46%) described the specific role of a moderator, who was to be a person not involved in the coercive event.

The duration of the intervention ranged from 10 to 45 minutes. Some articles emphasized the importance of flexibility and the possibility of conducting multiple sessions if needed. Eight articles (62%) reported a recommended time point for the implementation of the intervention after the coercive event. Recommendations ranged from 48 hours to 3 days but reported data from the experimental studies showed that interventions were delivered up to 118 days later. In some interventions (n = 3; 23%), patients themselves were allowed to choose the preferred point of time. More than half of the articles (n = 7; 54%) mentioned a training of the health care professionals before the tool was used in practice. This included presentations and opportunities to consult with the researchers (40) or prompt cards outlining the principles and process for conducting the debriefing intervention (13).

### 3.4 SDM elements

**Table 4** shows the SDM elements present in each post-coercion intervention. There was no intervention in which all SDM elements were clearly present. Three elements of SDM were present in all interventions, in some cases at least partially: 1.) definition and explanation of the health care problem, 4.) the clarification of patient’s values and preferences, and 8.) a final decision or explicit deferral of the decision. Almost all interventions (n = 12; 92%) included the SDM element of 2.) presenting and reviewing existing options.

**Table 4:** SDM elements of the identified post-coercion debriefing interventions.

Authors, year	SDM 1	SDM 2	SDM 3	SDM 4	SDM 5	SDM 6	SDM 7	SDM 8	SDM 9
Asikainen et al., 2023 (45)	X	X	–	X	(X)	–	(X)	X	–
Dike et al., 2021 (32)	X	X	–	X	–	–	–	X	–
Drack-Schönenberger et al., 2016 (46)	X	(X)	–	X	(X)	–	X	X	–
Freier, 2022 (39)	X	X	–	(X)	–	–	(X)	X	–
Goulet et al., 2018 (40)	X	X	(X)	X	(X)	–	–	(X)	–
Hammervold et al., 2020 (35)	X	X	–	X	–	–	–	X	–
Hammervold et al., 2022 (36)	X	X	–	X	(X)	–	–	X	–
Lorem et al., 2014 (42)	X	X	X	X	(X)	X	X	X	–
Mahler et al., 2021 (15)	X	X	X	X	(X)	–	X	X	X
Tinland et al., 2022 (47)	X	–	–	X	–	–	–	X	–
Whitecross et al., 2013 (13)	X	(X)	–	X	X	–	–	X	–
Wullschleger et al., 2019 (37)	X	X	X	X	(X)	–	X	X	X
Wullschleger et al., 2021 (38)	X	X	X	X	(X)	–	X	X	X

X = present; (X) = unclear/partially present; - = not present.

SDM = Shared decision-making;

SDM 1 = define and/or explain the healthcare problem; SDM 2 = present options; SDM 3 = discuss pros and cons (benefits, risks, costs); SDM 4 = clarify patient values and preferences; SDM 5 = discuss patient ability and self-efficacy; SDM 6 = present what is known and make recommendations; SDM 7 = check and clarify the patient’s understanding; SDM 8 = make or explicitly defer a decision; SDM 9 = arrange follow-up.

The other SDM elements were less common, and there was more uncertainty about whether these elements were present, particularly regarding 5.) discussing of patient ability and self-efficacy (n = 9; 69%), 7.) checking and clarifying of the patient’s understanding (n = 7; 54%), and 3.) discussing the pros and cons (n = 5; 38%). The SDM element mentioned in only one article (8%) was 6.) presenting of professional knowledge and recommending of options.

## 4 Discussion

### 4.1 Overview of findings

The aim of this scoping review was to examine general characteristics and SDM elements in post-coercion debriefing interventions in psychiatry. Our findings provide additional insights into the extent to which the concept of SDM is already being used in this context.

The main finding from the analysis of the general characteristics was a great variety regarding the participants, the proposed time point of application and the content of the intervention across the articles. As national and international guidelines provide little information on how to conduct debriefings, lack of standards may explain this observation. In addition, the diversity may be well in line with the conclusion of previous research, which also reported on heterogeneity in debriefing intervention elements and the underlying theoretical approaches (10, 15, 41).

Another important finding is the limited extent of SDM elements used in the debriefing interventions. Our review of the literature documents no study that describes a debriefing intervention that fulfills all elements of the analyzed SDM concept. Furthermore, the concept itself is only mentioned by name in one study (42). Interventions may include general qualities of the SDM concept, such as patient participation or the involvement of at least two people, but not necessarily all the essential elements as proposed by Makoul and Clayman’s integrative model of SDM (17).

The heterogeneity in general and in SDM elements makes it more difficult to identify effective elements for the construction of a new tool. However, the interventions described below were particularly interesting because of the extensive description of general intervention characteristics and/or the variety of SDM elements they included. These two debriefing interventions could serve as a useful starting point for what an SDM intervention after coercive measures might look like:

Mahler et al. (15) proposed a debriefing intervention as part of a more complex concept for patient-oriented psychiatry, the ‘Weddinger Modell’ (11). The intervention is attended by the patient, a contact person of the patient (optional) and a care provider involved in the coercive event. A member of the team who was not involved in the coercive event facilitates the 30 to 45-minute guideline-based intervention. The patient determines the preferred time for the debriefing intervention. The debriefing intervention fulfills all SDM elements except for the element 6.) presentation of professional knowledge and recommendation of options. The intervention was also used by other identified literature. A theoretical article by Freier suggested a slightly adapted wording of the intervention (39). Wullschleger et al. investigated the feasibility and effectiveness of the debriefing intervention in a qualitative pilot study (37) and an RCT (38). According to their results, the debriefing intervention was well received and could contribute to reducing the burden of PTSD symptoms in patients with psychotic disorders following a coercive event in an acute setting.

Lorem et al. (42) presented an approach that fulfills all SDM elements except for the element 9.) follow-up, although this was mentioned as a good addition for the future. It is also the only article that clearly included the SDM element 6.) presentation of professional knowledge and the recommendation of options. However, the study focuses on the narrow context of treatment planning for antipsychotic medication without discussing the preceding coercive event in detail and without providing further details on general elements of the intervention.

The findings also support the notion that the debriefing process can include different steps, with partly different goals. Most debriefing interventions focused on a retrospective evaluation of the coercive event, aiming at transparency and emotional relief. In a second step, some interventions moved on to the planning for future care and dealing with possible future coercive events, focusing on preventive goals such as reducing coercive measures in the future. The information and insights from the first step provide the basis for the decision-making process. Both steps may also serve the same goals, such as strengthening the therapeutic relationship. The concept of SDM can be applied mainly in this second step, as this part of the process is aimed at further treatment planning. Therefore, a structured decision aid might be useful to ensure that all elements of SDM are included. This leads to the conclusion that the processual nature of debriefing interventions involving SDM, with different steps and objectives, should be adequately addressed in a new intervention that includes an analysis tool and a structured decision aid.

The review did not identify any digital application of post-coercion debriefing interventions. It remains unclear to what extent elements used in analog applications can be directly adapted.

### 4.2 Strengths and limitations

A strength of the present review is the systematic literature search with broad search blocks and inclusion criteria that captured a wide range of scientific articles addressing the topic. The overview of general and SDM elements used in the interventions provides a helpful starting point for the development of a new digital DA for debriefing in the context of coercive measures.

However, there may be other studies that addressed the topic of SDM peripherally or through proxies that fell outside the scope of our search, despite the use of many keywords. Most articles discussed issues related to patient participation in decision making at debriefing interventions but did not explicitly aim to adapt the principle of SDM.

The scoping review does not address the quality of evidence by design and therefore provides only a descriptive summary of the available articles on the topic. For the construction of an effective digital decision aid, it will be important to build on elements of evidence-based interventions and to adapt them appropriately. Therefore, it can be recommended to focus mainly on elements of the interventions that have been tested for effectiveness in RCTs.

In addition, we should draw on the expertise of design research in the construction of a digital tool, as no further evidence on this point can be drawn from this review.

## 5 Conclusions

In general, more research is needed to systematically examine the implementation and clinical effectiveness of post-coercion debriefing interventions. The variety of study designs in this scoping review, ranging from purely theoretical descriptions to RCTs, poses a challenge for the interpretation of the existing evidence, but allows insights into different aspects of interest.

In future studies, it is important to rigorously evaluate different types of debriefing interventions to identify therapeutically effective elements. A more robust understanding of the specific facets of decision making that are most important to patients and health care providers would aid in the development of debriefing interventions. Studies using qualitative methods or mixed-methods approaches may provide better means of elucidating these aspects than quantitative studies.

In addition, it may be essential for further research to include considerations of the impact of psychiatric patients’ decision-making capacities. It has been suggested that most psychiatric patients, including those with severe illnesses such as schizophrenia, may have the capacity to participate in medical decisions related to their illness (43). Nevertheless, patients’ potentially diminished capacity must be addressed. Ensuring a nuanced understanding of SDM in these cases may enhance the development of person-centered decision-making processes (44).

## Data Availability

The original contributions presented in the study are included in the article/**Supplementary Material**. Further inquiries can be directed to the corresponding author.
