# Supplementary material for: Shared decision-making in post-coercion debriefing interventions in psychiatry – a scoping review
Source: Front Psychiatry. 2024 Sep 27;15:1446619. doi: 10.3389/fpsyt.2024.1446619 (PMC11466772; doi:10.3389/fpsyt.2024.1446619)
Supplement: Supplementary file 1 [file Table1.docx]

**Supplementary Material A: Search strategy**

*MEDLINE (PubMed)*

*Search conducted in Tab "Advanced Search"*

("decision making*"[MeSH Terms] OR "clinical decision making"[MeSH Terms] OR "debriefing*"[Title/Abstract] OR "follow-up decision making*"[Title/Abstract] OR "evaluation*"[Title/Abstract] OR "review*"[Title/Abstract] OR "post coercion review*"[Title/Abstract] OR "post incident review*"[Title/Abstract] OR "post event review*"[Title/Abstract] OR "post event analys*"[Title/Abstract])

AND

("decision making, shared"[MeSH Terms] OR "patient participation"[MeSH Terms] OR "patient-centered care"[MeSH Terms] OR "patient preference"[MeSH Terms] OR "shared decision making*"[Title/Abstract] OR "SDM"[Title/Abstract] OR "supported decision making*"[Title/Abstract] OR "patient decision making*"[Title/Abstract] OR "patient involvement*"[Title/Abstract] OR "patient empowerment*"[Title/Abstract] OR "patient activation*"[Title/Abstract] OR "patient engagement*"[Title/Abstract] OR "patient centeredness"[Title/Abstract] OR "client participation"[Title/Abstract] OR "cooperation*"[Title/Abstract] OR "share*"[Title/Abstract] OR "prefer*"[Title/Abstract] OR "patient centered*"[Title/Abstract] OR "patient-focused"[Title/Abstract])

AND

("mental disorders"[MeSH Terms] OR "mental health"[MeSH Terms] OR "mentally ill persons"[MeSH Terms] OR "psychiatry"[MeSH Terms] OR "hospitals, psychiatric"[MeSH Terms] OR "psychiatric department, hospital"[MeSH Terms] OR "mental health services"[MeSH Terms] OR "mental disorder*"[Title/Abstract] OR "psychiatric illness*"[Title/Abstract] OR "psychiatric disease*"[Title/Abstract] OR "mental illness*"[Title/Abstract] OR "psychiatric disorder*"[Title/Abstract] OR "behavior disorder*"[Title/Abstract] OR "psychiatric diagnos*"[Title/Abstract] OR "abnormal mental state*"[Title/Abstract] OR "disordered mental state*"[Title/Abstract] OR "disturbed mental state*"[Title/Abstract] OR "insanity"[Title/Abstract] OR "mental abnormalit*"[Title/Abstract] OR "mental change*"[Title/Abstract] OR "mental confusion*"[Title/Abstract] OR "mental defect"[Title/Abstract] OR "mental disease*"[Title/Abstract] OR "mental disturbance*"[Title/Abstract] OR "mental insufficiency"[Title/Abstract] OR "mental symptom*"[Title/Abstract] OR "neurodevelopmental disorder*"[Title/Abstract] OR "neuropsychiatric disease*"[Title/Abstract] OR "neuropsychiatric disorder*"[Title/Abstract] OR "psychiatric disease*"[Title/Abstract] OR "psychiatric disorder*"[Title/Abstract] OR "psychiatric illness*"[Title/Abstract] OR "psychiatric symptom*"[Title/Abstract] OR "psychic disease*"[Title/Abstract] OR "psychic disorder*"[Title/Abstract] OR "psychic disturbance*"[Title/Abstract] OR "psychologic disorder*"[Title/Abstract] OR "psychologic disturbance*"[Title/Abstract] OR "psychopathology"[Title/Abstract] OR "mental health"[Title/Abstract] OR "mental hygiene"[Title/Abstract] OR "mentally ill person*"[Title/Abstract] OR "mentally ill"[Title/Abstract] OR "mental patient*"[Title/Abstract] OR "psychiatric patient*"[Title/Abstract] OR "psychiatry patient*"[Title/Abstract] OR "psychiatric clinic*"[Title/Abstract] OR "psychiatric hospital*"[Title/Abstract] OR "mental institution*"[Title/Abstract] OR "mental hospital*"[Title/Abstract] OR "asylum*"[Title/Abstract] OR "mental hospital*"[Title/Abstract] OR "mental institution*"[Title/Abstract] OR "psychiatric hospital*"[Title/Abstract] OR "psychiatric institution*"[Title/Abstract] OR "mental health clinic*"[Title/Abstract] OR "mental health unit*"[Title/Abstract] OR "mental institute*"[Title/Abstract] OR "mental health care services"[Title/Abstract] OR "psychiatric center*"[Title/Abstract] OR "mental health care"[Title/Abstract] OR "psychiatric care"[Title/Abstract] OR "psychiatrist*"[Title/Abstract] OR "mental health personnel"[Title/Abstract])

AND

("coercion"[MeSH Terms] OR "commitment of mentally Ill"[MeSH Terms] OR "involuntary commitment"[MeSH Terms] OR "restraint, physical"[MeSH Terms] OR "mentally ill commitment*"[Title/Abstract] OR "outpatient commitment*"[Title/Abstract] OR "civil commitment*"[Title/Abstract] OR "mental health commitment*"[Title/Abstract] OR "coercive treatment*"[Title/Abstract] OR "compulsory commitment*"[Title/Abstract] OR "compulsory hospitalization*"[Title/Abstract] OR "compulsory psychiatric admission*"[Title/Abstract] OR "involuntary admission*"[Title/Abstract] OR "involuntary hospitalization*"[Title/Abstract] OR "involuntary psychiatric commitment*"[Title/Abstract] OR "involuntary psychiatric treatment*"[Title/Abstract] OR "involuntary treatment*"[Title/Abstract] OR "legal commitment*"[Title/Abstract] OR "psychiatric commitment*"[Title/Abstract] OR "restraint*"[Title/Abstract] OR "mechanical restraint*"[Title/Abstract] OR "physical restraint*"[Title/Abstract] OR "seclusion"[Title/Abstract] OR "compulsi*"[Title/Abstract] OR "coerc*"[Title/Abstract] OR "involuntar*"[Title/Abstract] OR "not voluntary"[Title/Abstract] OR "against will"[Title/Abstract] OR "forced"[Title/Abstract])

*EMBASE (ELSEVIER)*

*Search conducted in Tab "Advanced Search" (Keyword) with removed check mark at "Map Term to Subject Heading"*

('decision making'/exp OR 'clinical decision making'/exp OR 'debriefing*':ti,ab OR 'follow up decision making*':ti,ab OR 'evaluation*':ti,ab OR 'review*':ti,ab OR 'post coercion review*':ti,ab OR 'post incident review*':ti,ab OR 'post event review*':ti,ab OR 'post event analys*':ti,ab)

AND

('shared decision making'/exp OR 'shared decision making':ti,ab OR 'patient participation'/exp OR 'patient-centered care':ti,ab OR 'patient preference':ti,ab OR 'SDM':ti,ab OR 'supported decision making*':ti,ab OR 'patient decision making'/exp OR 'patient involvement*':ti,ab OR 'patient empowerment'/exp OR 'patient activation*':ti,ab OR 'patient engagement'/exp OR 'patient centeredness':ti,ab OR 'client participation':ti,ab OR 'cooperation*':ti,ab OR 'share*':ti,ab OR 'prefer*':ti,ab OR 'patient centered*':ti,ab OR 'patient-focused':ti,ab)

AND

('mental disorder*':ti,ab OR 'mental health'/exp OR 'mentally ill person*':ti,ab OR 'psychiatry'/exp OR 'psychiatric hospitals':ti,ab OR 'psychiatric department':ti,ab OR 'mental health service'/exp OR 'psychiatric illness*':ti,ab OR 'psychiatric disease*':ti,ab OR 'mental illness*':ti,ab OR 'psychiatric disorder*':ti,ab OR 'behavior disorder*':ti,ab OR 'psychiatric diagnos*':ti,ab OR 'abnormal mental state*':ti,ab OR 'disordered mental state*':ti,ab OR 'disturbed mental state*':ti,ab OR 'insanity':ti,ab OR 'mental abnormalit*':ti,ab OR 'mental change*':ti,ab OR 'mental confusion*':ti,ab OR 'mental defect':ti,ab OR 'Mental Disease'/exp OR 'mental disturbance*':ti,ab OR 'mental insufficiency':ti,ab OR 'mental symptom*':ti,ab OR 'neurodevelopmental disorder*':ti,ab OR 'neuropsychiatric disease*':ti,ab OR 'neuropsychiatric disorder*':ti,ab OR 'psychiatric disease*':ti,ab OR 'psychiatric disorder*':ti,ab OR 'psychiatric illness*':ti,ab OR 'psychiatric symptom*':ti,ab OR 'psychic disease*':ti,ab OR 'psychic disorder*':ti,ab OR 'psychic disturbance*':ti,ab OR 'psychologic disorder*':ti,ab OR 'psychologic disturbance*':ti,ab OR psychopathology:ti,ab OR 'mental health':ti,ab OR 'mental hygiene':ti,ab OR 'mentally ill person*':ti,ab OR 'mentally ill':ti,ab OR 'mental patient'/exp OR 'psychiatric patient*':ti,ab OR 'psychiatry patient*':ti,ab OR 'psychiatric clinic*':ti,ab OR 'psychiatric hospital*':ti,ab OR 'mental institution*':ti,ab OR 'mental hospital'/exp OR 'mental hospital*':ti,ab OR 'asylum*':ti,ab OR 'mental institution*':ti,ab OR 'psychiatric hospital*':ti,ab OR 'psychiatric institution*':ti,ab OR 'mental health clinic*':ti,ab OR 'mental health unit*':ti,ab OR 'mental institute*':ti,ab OR 'mental health care service*':ti,ab OR 'psychiatric center*':ti,ab OR 'mental health care'/exp OR 'mental health care':ti,ab OR 'psychiatric care':ti,ab OR 'Psychiatrist'/exp OR 'psychiatrist*':ti,ab OR 'mental health personnel'/exp OR 'mental health personnel':ti,ab)

AND

('coercion'/exp OR 'commitment of mentally ill':ti,ab OR 'involuntary commitment'/exp OR 'physical restraint'/exp OR 'mentally ill commitment*':ti,ab OR 'outpatient commitment*':ti,ab OR 'civil commitment*':ti,ab OR 'mental health commitment':ti,ab OR 'coercive treatment*':ti,ab OR 'compulsory commitment*':ti,ab OR 'compulsory hospitalization*':ti,ab OR 'compulsory psychiatric admission*':ti,ab OR 'involuntary admission*':ti,ab OR 'involuntary hospitalization*':ti,ab OR 'involuntary psychiatric commitment*':ti,ab OR 'involuntary psychiatric treatment*':ti,ab OR 'involuntary treatment*':ti,ab OR 'legal commitment*':ti,ab OR 'psychiatric commitment*':ti,ab OR 'restraint*':ti,ab OR 'mechanical restraint*':ti,ab OR 'physical restraint*':ti,ab OR 'seclusion’:ti,ab OR 'compulsi*':ti,ab OR 'coerc*':ti,ab OR 'involuntar*':ti,ab OR 'not voluntary':ti,ab OR 'against will' OR 'forced':ti,ab)

*PsychINFO (Ovid)*

*Search conducted in Tab "Advanced Search"*

(exp "decision making"/ OR "clinical decision making".ti,ab. OR debriefing*.ti,ab. OR "follow up decision making*".ti,ab. OR evaluation*.ti,ab. OR review*.ti,ab. OR "post coercion review*".ti,ab. OR "post incident review*".ti,ab. OR "post event review*".ti,ab. OR "post event analys*".ti,ab.)

AND

("patient participation".ti,ab. OR "patient centered care".ti,ab. OR "patient preference".ti,ab. OR "shared decision making*".ti,ab. OR "SDM".ti,ab OR "supported decision making*".ti,ab. OR "patient decision making*".ti,ab. OR "patient involvement*".ti,ab. OR "patient empowerment*".ti,ab. OR "patient activation*".ti,ab. OR "patient engagement*".ti,ab. OR "patient centeredness".ti,ab. OR "client participation".ti,ab. OR exp "client participation"/ OR "cooperation*".ti,ab. OR "share*".ti,ab. OR "prefer*".ti,ab. OR "patient centered*".ti,ab. OR "patient focused".ti,ab.)

AND

(exp "mental disorders"/ OR exp "mental health"/ OR "mentally ill persons".ti,ab. OR exp psychiatry/ OR "hospitals, psychiatric".ti,ab. OR "psychiatric department, hospital".ti,ab. OR "mental health services".ti,ab. OR "mental disorder*".ti,ab. OR "psychiatric illness*".ti,ab. OR "psychiatric disease*".ti,ab. OR "mental illness*".ti,ab. OR "psychiatric disorder*".ti,ab. OR "behavior disorder*".ti,ab. OR "psychiatric diagnos*".ti,ab. OR "abnormal mental state*".ti,ab. OR "disordered mental state*".ti,ab. OR "disturbed mental state*".ti,ab. OR "insanity ".ti,ab. OR "mental abnormalit*".ti,ab. OR "mental change*".ti,ab. OR "mental confusion*".ti,ab. OR "mental defect".ti,ab. OR "mental disease*".ti,ab. OR "mental disturbance*".ti,ab. OR "mental insufficiency".ti,ab. OR "mental symptom*".ti,ab. OR "neurodevelopmental disorder*".ti,ab. OR "neuropsychiatric disease*".ti,ab. OR "neuropsychiatric disorder*".ti,ab. OR "psychiatric disease*".ti,ab. OR "psychiatric disorder*".ti,ab. OR "psychiatric illness*".ti,ab. OR "psychiatric symptom*".ti,ab. OR "psychic disease*".ti,ab. OR "psychic disorder*".ti,ab. OR "psychic disturbance*".ti,ab. OR "psychologic disorder*".ti,ab. OR "psychologic disturbance*".ti,ab. OR exp "psychopathology "/ OR "mental health".ti,ab. OR "mental hygiene".ti,ab. OR "mentally ill person*".ti,ab. OR "mentally ill".ti,ab. OR "mental patient*".ti,ab. OR "psychiatric patient*".ti,ab. OR exp "psychiatric patients"/ OR "psychiatry patient*".ti,ab. OR "psychiatric clinic*".ti,ab. OR "psychiatric hospital*".ti,ab. OR "mental institution*".ti,ab. OR "mental hospital*".ti,ab. OR asylum*.ti,ab. OR "mental hospital*".ti,ab. OR "mental institution*".ti,ab. OR "psychiatric hospital*".ti,ab. OR "psychiatric institution*".ti,ab. OR "mental health clinic*".ti,ab. OR "mental health unit*".ti,ab. OR "mental institute*".ti,ab. OR "mental health care services".ti,ab. OR "psychiatric center*".ti,ab. OR "mental health care".ti,ab. OR "psychiatric care".ti,ab. OR psychiatrist*.ti,ab. OR "mental health personnel".ti,ab. OR exp "mental health personnel"/)

AND

(exp "coercion"/ OR "commitment of mentally ill".ti,ab. OR "involuntary commitment" OR exp "restraint, physical"/ OR "mentally ill commitment*".ti,ab. OR "outpatient commitment*".ti,ab. OR "civil commitment*".ti,ab. OR "mental health commitment".ti,ab. OR "coercive treatment*".ti,ab. OR "compulsory commitment*".ti,ab. OR "compulsory hospitalization*".ti,ab. OR "compulsory psychiatric admission*".ti,ab. OR "involuntary admission*".ti,ab. OR "involuntary hospitalization*".ti,ab. OR "involuntary psychiatric commitment*".ti,ab. OR "involuntary psychiatric treatment*".ti,ab. OR exp "involuntary treatment"/ OR "involuntary treatment*".ti,ab. OR "legal commitment*".ti,ab. OR "psychiatric commitment*".ti,ab. OR restraint*.ti,ab. OR "mechanical restraint*".ti,ab. OR "physical restraint*".ti,ab. OR seclusion.ti,ab. OR exp "patient seclusion"/ OR compulsi*.ti,ab. OR coerc*.ti,ab. OR involuntar*.ti,ab. OR "not voluntary".ti,ab. OR "against will".af. OR forced.ti,ab.)

*WEB OF SCIENCE*

*Search conducted in "Basic Search" (TOPIC)*

("decision making*" OR "clinical decision making" OR "debriefing*" OR "follow up decision making*" OR evaluation* OR review* OR "post coercion review*" OR "post incident review*" OR "post event review*" OR "post event analys*")

AND

("decision making shared" OR "patient participation" OR "patient-centered care" OR "patient preference" OR "shared decision making*" OR "SDM" OR "supported decision making*" OR "patient decision making*" OR "patient involvement*" OR "patient empowerment*" OR "patient activation*" OR "patient engagement*" OR "patient centeredness" OR "client participation" OR cooperation* OR share* OR prefer* OR "patient centered*" OR "patient-focused")

AND

("mental disorders" OR "mental health" OR "mentally ill persons" OR psychiatry OR "hospitals, psychiatric" OR "psychiatric department, hospital" OR "mental health services" OR "mental disorder*" OR "psychiatric illness*" OR "psychiatric disease*" OR "mental illness*" OR "psychiatric disorder*" OR "behavior disorder*" OR "psychiatric diagnos*" OR "abnormal mental state*" OR "disordered mental state*" OR "disturbed mental state*" OR insanity OR "mental abnormalit*" OR "mental change*" OR "mental confusion*" OR "mental defect" OR "mental disease*" OR "mental disturbance*" OR "mental insufficiency" OR "mental symptom*" OR "neurodevelopmental disorder*" OR "neuropsychiatric disease*" OR "neuropsychiatric disorder*" OR "psychiatric disease*" OR "psychiatric disorder*" OR "psychiatric illness*" OR "psychiatric symptom*" OR "psychic disease*" OR "psychic disorder*" OR "psychic disturbance*" OR "psychologic disorder*" OR "psychologic disturbance*" OR psychopathology OR "mental health" OR "mental hygiene" OR "mentally ill person*" OR "mentally ill" OR "mental patient*" OR "psychiatric patient*" OR "psychiatry patient*" OR "psychiatric clinic*" OR "psychiatric hospital*" OR "mental institution*" OR "mental hospital*" OR asylum* OR "mental hospital*" OR "mental institution*" OR "psychiatric hospital*" OR "psychiatric institution*" OR "mental health clinic*" OR "mental health unit*" OR "mental institute*" OR "mental health care services" OR "psychiatric center*" OR "mental health care" OR "psychiatric care" OR psychiatrist* OR "mental health personnel")

AND

("coercion" OR "commitment of mentally ill" OR "involuntary commitment" OR "restraint, physical" OR "mentally ill commitment*" OR "outpatient commitment*" OR "civil commitment*" OR "coercive treatment*" OR "compulsory commitment*" OR "compulsory hospitalization*" OR "compulsory psychiatric admission*" OR "involuntary admission*" OR "involuntary hospitalization*" OR "involuntary psychiatric commitment*" OR "involuntary psychiatric treatment*" OR "involuntary treatment*" OR "legal commitment*" OR "psychiatric commitment*" OR restraint* OR "mechanical restraint*" OR "physical restraint*" OR "seclusion" OR "compulsi*" OR "coerc*" OR "involuntar*" OR "not voluntary" OR "against will" OR "forced")

Advanced Search Error: Your search exceeded the maximum number of terms allowed in one field (50 terms for "All Fields" and 16,000 terms for others, separated by Boolean operators). Please shorten your query.

- Select “topic” instead of “all fields”
